# Spring Dangers

**Published:** 1873-04

**Authors:** 


### HALL'S

### JOURNAL OF HEALTH

Vol. XX.—APRIL, 1873.—No. IV.

### SPRING DANGERS.

March, April, and May are more fruitful of sickness and death than the other months of the year; not so many die as in July, August, and September, but there is more disease, arising mainly from

Sudden changes of the weather;

Injudicious changes in clothing, and

Errors in eating.

The above causes of sickness, suffering, and death are avoidable; hence we are the authors of our own sufferings, which are irrationally attributed too often to the "mysterious dispensations of Providence." The difference to the reader between attention and neglect to some of the following suggestions may be vigorous health or a shroud before midsummer.

In March there is a searching rawness and dampness in the atmosphere which chills the blood in such a way as to lay the foundation for a multitude of forms of colds, fevers, and inflammations.

In April the atmosphere is not so much saturated with dampness, but the fires are too early put out, and the winter clothing is too soon laid aside, in whole or in part.

In the beautiful May the mornings and evenings are damp and chilly; the warmth of midday tempts many to lay aside the warmer clothing, and this lighter clothing, together with the absence of fires, makes a difference sometimes of twenty, thirty, or even forty degrees.

But, in addition to these, there is another cause of disease, almost universal, prevailing through all the three months. No one thinks of building as fierce fires in the spring time as in midwinter, because there is not the same necessity for artificial heat, the external air is not near so cold, but we kindle as great an internal heat, which is excessive, and that is fever, and fever is followed with cold within any twenty-four hours as certainly as a pendulum, carried to the right, if let go, will swing to the left; one is the reaction of the other, a fixed law of our being. If the system was kept in an equable condition of warmth within and without, we would have neither fevers nor colds, chills nor inflammations.

We eat for two reasons: to sustain and to warm. A large portion of our meats and butter and bread is composed of the warming principle, carbon, the province of which is to produce animal heat; hence, if we eat as much in the spring as in winter there is inevitably too much internal fire, and that is fever; and here comes in the wise arrangement of the

ROMAN CATHOLIC CHURCH, which requires that very little meat shall be eaten for a month or more; less substantial food is allowed, making the

LENTEN SEASON

a duty and a blessing; just as many observances under the Mosaic Economy, while they indicated an obedient spirit, brought with them temporal blessings, and that greatest of them all, good health; for, on close inspection, it will be found that these were promotive of cleanliness, temperance, and industry, than which there are not three greater medicaments within all human knowledge; and so it is in the later, the Christian dispensation. The precepts of the New Testament bring with them the blessings of "the life that now is, and of that which is to come," for it is commanded, "If any would not work, neither should he eat." "Be ye temperate in all things." "If any man provide not for his own house he is worse than an infidel." "Diligent in business." "Long-suffering, gentleness, goodness, temperance; forbearing one another in love." "Be content with your wages." "Oppress not the hireling in his wages," by failing to pay promptly, as soon as these wages are due. "Owe no man anything;" while it is "utterly a fault for one to go to law with another." Suppose a community should be organized under constitutional principles such as these, as general moral obligations, there would be forthwith a happiness, a thrift, an elevation, a health, and a bodily vigor not hitherto known among any people under the sun. This is not an unauthorized episode; an unwarrantable digression; for these things have been brought in to give encouragements to abstinence from strong food in the spring time as a duty and a wisdom.

In the spring everybody gets weaker because the weather is warmer, and yet we eat with the winter's appetite, until nature begins to take it away; then we begin to think that something is the matter, that we are going to get sick; but, instead of being content to diminish the quantity and quality of our food in proportion to the diminishing appetite, we begin to stimulate that appetite by "tonics" and ' bitters," meaning thereby

WHISKEY IN DISGUISE; for it is scarcely possible to find a tonic or a bitter or an appetizer of which alcohol is not a large constituent; if they do whet the appetite they are injurious just in proportion as they do it; it is fighting against nature; for, while she is endeavoring to moderate our desire för eating, we are trying to increase it; she aims to diminish the amount of fuel to supply the internal furnace; we try to increase it. In fact she goes further, absolutely changes the appetite. In February we revel on pork and fat and sweets and buckwheat cakes and corn bread, which are almost wholly "carbon;" we all know them to be "heating;" and as spring comes on we "relish greens" and spinage, the early vegetables and fruits and berries, which we all know to be "cooling" with their delicious acidities; how we delight in cabbages and turnip tops, and other green things which are eaten with vinegar, and turn away from pastries and puddings and doughnuts, and other forms of fats and sweets!

If, in the spring, we eat with the appetite of winter or goad ourselves to it with tonics, the inevitable result in all cases, infallibly, is that there is too much internal heat, meaning fever, followed by biliousness with its long catalogue of "ails," ending in dysenteries, diarrheas and insufferable debilities.

Families make a great mistake, especially in the country, in early dispensing with fires. Where there are children there should be a good blazing

FIRE ON THE HEARTH, at least in one room in the house, all day, until the first of May, and during May until after breakfast, to be kindled up at sun-down. The instinct of the children will drive them to that room, and when they cease to gather around that fire, mornings and evenings, then, and not till then, ought fires to cease for the summer.

### CHANGING CLOTHING.

Health is often lost, and sometimes life itself, by laying aside winter clothing too early. Laying flannels aside in the spring is a most pernicious practice. They are as necessary in July as in January. We can better do without woolens next the skin in midwinter than in midsummer. We do not get over heated in winter; we do in summer; and the most frequent exciting cause of coughs, colds, and consumption is a rapid falling of the temperature of the body. All are familiar with the fact that a sudden checking of perspiration is always dangerous; very little exercise causes us to perspire in summer, and a very slight draught of air checks the perspiration; hence, eminent French physicians have stated, after a long series of observations, that colds taken in summer excite the most incurable forms of consumption. White woolen flannel is a most efficient guard against these sudden changes, because it keeps the heat of the body in while it repels the excessive heat from without; it conveys the water of perspiration to its outside, while the surface next the skin is drier. We all know that silk, cotton, and linen next the skin get saturated with water, and if, for an instant, the slightest draft of air gets between the skin and the material there is a charnel-like chill when that material touches the skin again, reminding us of

THE DAMPS OF DEATH.

The rule should be to wear white woolen flannel next the skin all the year round; thick in winter, a little thinner in April, a gauze material on the first day of July; on the first of October resume what was laid aside in July; on the first of December put on the thickest, extending to ankles and wrists.

These rules of change are especially necessary to all old people, to all invalids and young children; day laborers and all out-door workers would be incalculably benefited by the same observances. These suggestions are of an importance which would justify their being read in the spring and fall to every household until the youngest child has passed the threshold to return no more as a member of the family.

The reader's attention is directed to several other articles in this number, embodying the same ideas, but presented in a different light with the express purpose of having the subject more thoroughly understood and impressed on the mind, in the hope that the lessons may be remembered by the present reader for life, and that they may be taught to children and children's children, for they are true and practical and useful, and will be for all time; the page on "Checking Perspiration," for example.

### ACCIDENTS.

Another word for carelessness, but their consequences may be antagonized in many cases if we were to acquaint ourselves beforehand with the means of treating them; and if further we should show and explain to our children how to meet them, giving the reason for the same. Not long ago a little girl skated into an air hole and was in peril of drowning; every time she made an effort to crawl out on the ice it broke, and she fell back into the water. A companion, only twelve years old, crawled on all fours, flat on her stomach, and had another girl to do the same behind her, but catching firmly hold of her ankle; she made her way forward until her freezing and drowning companion could take hold of her hand, and then drew her out safely. When asked how she came to think of such a plan of procedure, she said she had heard father and mother talking about it a few days before. The philosophy and wisdom of the whole transaction can be studied out by the thoughtful reader.

### LONGFELLOW.

Multitudes of intelligent admirers sympathized with the great poet in the crowning sorrow of his life, when his accomplished wife was burned to death by her dress taking fire in the act of her sealing a letter with wax; a part of the flaming material having fallen on the gauzy fabric. The husband was in an adjoining room; at hearing her shrieks he was instantly at her side, but it was too late; she breathed the flame into her lungs, and all was over. If she had instantly thrown herself on the floor, and the husband, on his reaching the spot, had thrown a carpet or blanket, or even his coat, over her, she could not have been fatally burned.

The whole subject could be indelibly impressed on the minds of a family of children in ten minutes, thus: Clothe a doll with the finest muslin or paper, hold it upright and set the dress on fire; in an instant almost the head will be enveloped in flame. Clothe it again, set it on fire and instantly lay it on the side, and it will be seen that the face had not been touched, and instead of trying to put out the flame with the hands, throw over it any cotton material, the flame will be increased; then throw over it a woolen material, and it will be instantly put out.

Suppose again that you wanted to convince children of the injuriousness of

EATING TOO FAST, and the advantages of cutting up food in very small pieces and chewing it deliberately. Have two glasses, half filled with water; take a piece of ice and divide it equally; cut up one piece into pea-sized particles and put them in one glass; put the other piece in one lump into the other glass; note the time, stir each one with a spoon, thus showing how much longer it will require to melt the one lump than the many, dissolving, as they all do, from without, inwards. Then explain that the food in the stomach is dissolved from without, inwards; that the sooner it is dissolved the better, and the quicker it will be ready to be appropriated to the uses of the system; such an illustration could never be forgotten, and the whole philosophy of digestion would be ever thereafter fully comprehended. In similar ways could other important truths connected with health be impressed on the minds of the young, as may be seen in our last book, "Health at Home," or Hall's Family Doctor. The practical question is, ought we not to take some pains to instruct our children in at least some of the main points about preserving their health?—for without health life is a failure.

——o——

### PAINFUL AFFECTIONS.

PERSONS sometimes suffer a variety of pains, more or les permanent, without being able to trace out the cause, the bodily functions seeming to be in healthy action, and yet medicine has no efficiency towards their removal. Sometimes these pains come on at regular intervals; at others they appear to depend on the state of the weather, or time, of day, or age of the moon, or season of the year. Grateful and permanent relief, more or less prompt, is sometimes experienced on discarding the use of some accustomed stimulant, in the form of tobacco, snuff, cigars, tea, coffee-bitters, or liquors; all these things stimulate the brain, and if their use is continued the constant stimulation ends in inflammation, communicating itself to the whole nervous system, causing a diseased condition of every fibre of the human body.

In many cases a single stimulation of the brain or nervous system is a medication of value and virtue, but habitual stimulation is always pernicious, because it occasions increased action, an action which is contrary to nature, which nature never intended, never made provision for, hence must be hurtful. When a man feels the need of a stimulant, it really means that the system wants rest, the mind repose, and the brain sleep. Rest and sleep are the only safe stimulants, because they bring back to the system its natural power of action. Sleep and rest are, to the body and brain, what letting down the water gate is to the old fashioned mill at night; the water accumulates by the morning to be used during the day; so the brain power

